# Alterations in CX3CL1 Levels and Its Role in Viral Pathogenesis

**DOI:** 10.3390/ijms25084451

**Published:** 2024-04-18

**Authors:** Chunmei Zhang, Yusi Zhang, Ran Zhuang, Kun Yang, Lihua Chen, Boquan Jin, Ying Ma, Yun Zhang, Kang Tang

**Affiliations:** Department of Immunology, The Fourth Military Medical University, Xi’an 710032, China; hzcm1981@126.com (C.Z.); immuzys@fmmu.edu.cn (Y.Z.); fmmuzhr@fmmu.edu.cn (R.Z.); yangkunkun@fmmu.edu.cn (K.Y.); chenlh@fmmu.edu.cn (L.C.); immu_jin@fmmu.edu.cn (B.J.); merry_bg20@163.com (Y.M.)

**Keywords:** CX3CL1, CX3CR1, chemokine, viral infection

## Abstract

CX3CL1, also named fractalkine or neurotactin, is the only known member of the CX3C chemokine family that can chemoattract several immune cells. CX3CL1 exists in both membrane-anchored and soluble forms, with each mediating distinct biological activities. CX3CL1 signals are transmitted through its unique receptor, CX3CR1, primarily expressed in the microglia of the central nervous system (CNS). In the CNS, CX3CL1 acts as a regulator of microglia activation in response to brain disorders or inflammation. Recently, there has been a growing interest in the role of CX3CL1 in regulating cell adhesion, chemotaxis, and host immune response in viral infection. Here, we provide a comprehensive review of the changes and function of CX3CL1 in various viral infections, such as human immunodeficiency virus (HIV), SARS-CoV-2, influenza virus, and cytomegalovirus (CMV) infection, to highlight the emerging roles of CX3CL1 in viral infection and associated diseases.

## 1. Introduction

Viruses give rise to a range of serious diseases that still pose challenges to contemporary medicine in terms of prevention and treatment. This is mainly attributed to the excessive activation of immune cells such as macrophages (Mφs), lymphocytes, and dendritic cells (DCs) via multiple signaling pathways [1]. The chemokine family contains approximately 50 kinds of endogenous factors in humans and mice [2,3,4], which have been identified and classified into four different families, namely the CC, CXC, CX3C, and C subfamilies [5,6,7]. C-X3-C motif ligand 1 (CX3CL1) belongs to the CX3C subfamily and possesses the characteristics of both homeostatic chemokines and inflammatory chemokines [7,8,9]. CX3CL1 and its receptor CX3CR1 were discovered more than twenty years ago [10,11,12], and a large amount of evidence has emerged linking the CX3CL1–CX3CR1 axis to various diseases, such as atherosclerosis [13], allergic diseases [14], neurodegeneration [15], and cancers [16,17]. It is worth noting that CX3CL1 is a critical element in the development of virus infection and related diseases, facilitating the migration of immune cells to distant organs [18,19]. 

**Figure 1:**
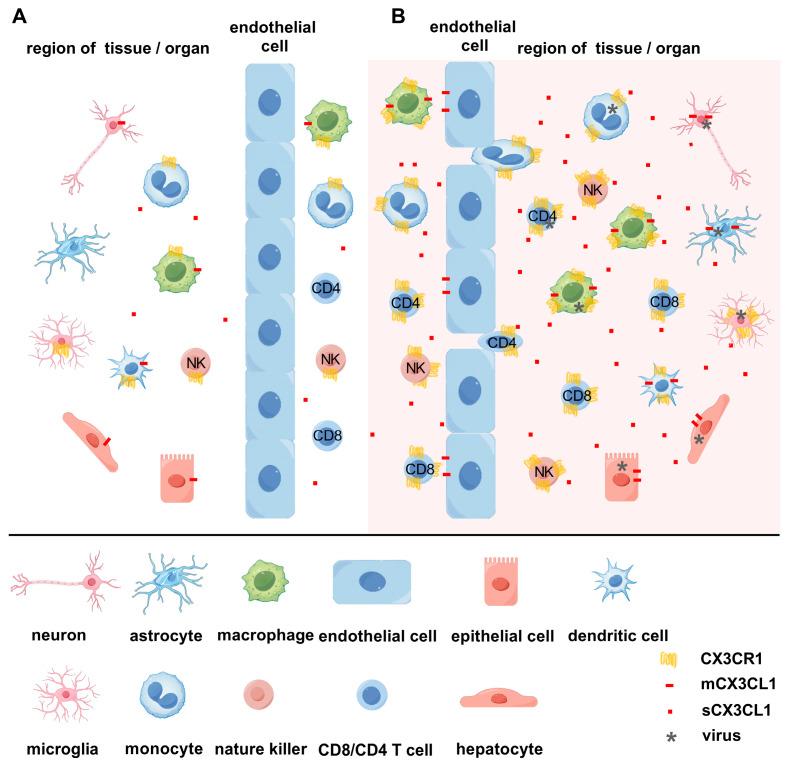
The expression levels of CX3CL1/CX3CR1 in the physiological state and after viral infection, as well as its adhesion and chemotactic functions. (**A**). Under physiological conditions, the membrane form of CX3CL1, expressed on cells like macrophages, epithelial cells, dendritic cells, and neurons, induces the adhesion of cells expressing CX3CR1. (**B**). During the process of most virus infection, especially in the acute stage, the expression of chemokine CX3CL1 is upregulated in activated and/or virus-infected macrophages, epithelial cells, dendritic cells, neurons, astrocytes, and endothelial cells, among others. The full-length mCX3CL1 serves as an adhesion molecule, and through CX3CR1, it promotes the retention of monocytes, nature killer cells, CD4 T cells, CD8 T cells, and other cells on epithelial or endothelial cells, and then plays a potential role in different viral infections. Part of CX3CL1 is cut into sCX3CL1 through ADAM10, ADAM17, or cathepsin S, mediating the migration of CX3CR1^+^ cells.

CX3CL1 presents in two forms: one is an 80–95 kDa glycoprotein with intracellular and transmembrane domains that are anchored to the membrane, and the other is cleaved by metalloproteinases A Disintegrin And Metalloprotease 10 (ADAM10), ADAM17 or cathepsin S [20], and released into the extracellular space to serve as a chemokine [21,22]. Both membrane-bound CX3CL1 (mCX3CL1) and soluble isoforms of CX3CL1 (sCX3CL1) can bind to CX3CR1 [23]. Compared with other chemokines and cytokines that can interact with various receptors, CX3CL1 binds only to one reported receptor, CX3CR1, to exert its biological effects. The distribution of CX3CL1 and CX3CR1 is shown in Figure 1 (by Figdraw 2.0). Since both CX3CL1 and CX3CR1 are expressed in various cells, it is not surprising that the CX3CL1–CX3CR1 axis may play a broader role, including stimulating cell proliferation [24,25] or migration [26,27], participating in angiogenesis [28,29], and promoting apoptosis [30]. Additionally, CX3CL1 can contribute to the recruitment of effector T cells to peripheral tissues and lymphoid organs [31], and participates in the adhesion between monocytes and endothelial cells [32]. There have been reports summarizing that most chemokines, such as CXCL2, CXCL9, and CXCL10, possess the ability to regulate the activation and migration of white blood cells, and control viral infections and the host defense functions of viral infections [33,34], but there is not yet a review summarizing the function of CX3CL1 in different viral infections.

In this article, we look again at the changes in CX3CL1 and its functional roles. Moreover, we review the accumulated evidence that the interactions of CX3CL1-CX3CR1 mediate significant events in viral infection, such as HIV, SARS-CoV-2, influenza virus, CMV, Dengue virus (DENV), ZIKA Virus (ZIKV), hepatitis B virus (HBV), viral infections, and associated diseases, especially through the recruitment of immune effector cells from the innate immune system via their chemotactic and adhesive properties.

## 2. CX3CL1 and Its Regulation of Chemotaxis and Adhesion

### 2.1. Structural Characteristics of CX3CL1 and Its Cellular Distribution

The human CX3CL1-encoding gene consists of three exons and is located on the long arm of the human chromosome 16q21, in the region of 57372490-57385044, functioning as both a chemoattractant and an adhesion molecule [35,36,37]. The respective genes are situated on chromosome 8 (8qC5) in mice and chromosome 19 (19p12) in rats [38,39]. The full-length CXC3L1 constitutes a 373-amino-acid type I transmembrane glycoprotein, comprising an extracellular N-terminal domain (aa 1–76), a mucin-like stalk (aa 77–317), a transmembrane alpha-helix (aa 318–336), and a short cytoplasmic tail (aa 337–373) [40,41]. For details of the schematic diagram of the structure, see the description in the previous literature [37]. It is highly expressed in Mφs, epithelial cells, DCs, renal mesangial cells, neurons, and smooth muscle cells, and can be induced in fibroblasts, endothelial cells, and astrocytes by several cytokines, such as IFN-γ, TNF-α, IL-1β, and TGF-β [42,43,44].

### 2.2. Functions of Membrane-Anchored CX3CL1

Recently, the role of the CX3CL1–CX3CR1 axis in inducing the chemotaxis and adhesion of leucocyte populations has been extensively studied by several research groups. The full-length mCX3CL1 functions as an adhesion molecule, promoting the retention of leucocytes to epithelial or endothelial cells through its G-protein-coupled, 7-transmembrane domain receptor CX3CR1 [45,46]. It is hypothesized that the mucin-like stalk within CX3CL1 is critical for the mechanism of CX3CL1–CX3CR1-mediated cell–cell adhesion [47,48]. Glycosylation ensures the accessibility of CX3CL1 to CX3CR1 buried in the membrane of the counter-adhesive cell, while the intracellular domain anchors CX3CL1 in the cell membrane [49]. mCX3CL1 can be significantly induced on primary endothelial cells by inflammatory cytokines and promote the firm adhesion of monocytes and T lymphocytes [9]. Moreover, mCX3CL1 acts as an adhesion molecule that influences neutrophil binding and adhesion and facilitates the penetration of immune cells through the vascular endothelium regardless of the integrin-related mechanism [7,50]. In the experiment of osteoblast binding, the addition of anti-CX3CL1 mAb results in the significant inhibition of osteoclast maturation, while the addition of recombinant CX3CL1 does not increase maturation. This indicates that the mCX3CL1-mediated adhesion plays an important role in the maturation of osteoclasts [50]. NK cells expressing CX3CR1 efficiently adhere to the full-length CX3CL1, but not to the truncated forms of the chemokine domain or mucin domain, indicating that mCX3CL1 functions as an adhesion molecule in the interaction between NK cells and endothelial cells in endothelial cell injury [51]. Given that mCX3CL1 functions as an adhesion protein to cells expressing CX3CR1, it causes immune system cells to remain on the vascular wall, close to the site of the inflammatory response, allowing these cells to migrate across the endothelium [52,53,54,55]. After entering the organization, mCX3CL1 can induce the proliferation of leukocytes like monocytes and CD16^+^ NK cells. However, there is no CX3CR1 expression in eosinophils and neutrophils, so CX3CL1 does not directly act on these cells [56].

### 2.3. Functions of Soluble CX3CL1

In both the physiological and pathological states, sCX3CL1 functions as a chemoattractant for CX3CR1^+^ cells [57,58,59,60] (Figure 1). In peripheral tissues, sCX3CL1 functions as a chemotactic peptide to form a concentration gradient in the extracellular matrix, attracting leukocytes to the sites of inflammation. Meanwhile, mCX3CL1 provides an adhesive function to capture circulating cells expressing CX3CR1 in endothelial cells, resulting in the migration of leukocytes to the tissues [61,62]. For instance, sCX3CL1 can regulate the adhesion and capture of circulating monocytes at the sites of atherogenesis [63]. Studies on cigarette smoke and lipopolysaccharide models of acute inflammation in transgenic *Cx3cr1*^gfp/gfp^ mice, as well as human endothelial cells and monocytes, demonstrated that sCX3CL1 mediated CX3CR1^+^ monocyte adhesion and migration [64]. These studies suggest that sCX3CL1 shows effective chemotactic activity for monocytes and T cells and modulates cell migration.

### 2.4. Regulation Mechanisms of CX3CL1 Expression

As research progresses, the regulatory cellular mechanisms of CX3CL1/CX3CR1-mediated cell adhesion and migration are continuously discovered. In innate immunity during viral infections, the binding of viral RNA to the helicase domains of retinoic-acid-inducible gene-1 and oligomerisation-domain-containing protein-like receptor-3 in myeloid cells activates the pro-inflammatory transcription factor nuclear factor (NF)-κB and the inflammasome [65,66,67], thereby inducing the release of CX3CL1 [65]. The stimulation of human umbilical arterial and venous endothelial cells with Ang-II increased CX3CL1 expression. The knockdown of *Nox5* with small interfering RNA or the pharmacological inhibition of extracellular signal-regulated kinases1/2, p38 mitogen-activated protein kinase, and nuclear factor-κB (NF-κB) also abolished the effect of tumor necrosis factor-α on Ang-II-induced CX3CL1 upregulation and mononuclear cell arrest [68]. CX3CL1 enhances the function of intercellular adhesion molecule-1 through the CX3CR1/PI3K/Akt/NF-κB signaling pathway and promotes the metastasis of osteosarcoma [69]. Following the adhesion associated with the interaction between CX3CL1 and CX3CR1, leukocytes are capable of enhancing adhesion in a directly selectin- and integrin-independent manner [70], or through synergistic effects with the activation and synthesis of other adhesion molecules [71,72,73]. In patients with chronic kidney disease, CD16^+^ monocytes enhance the STAT1 and NF-κB p65 phosphorylation of endothelial cells, and upregulate their expression of CX3CL1, IL-1β, CCL, CXCL, ICAM1, and VCAM1. This outlines a mechanism whereby the CX3CR1 dose-dependently modulates monocyte-contact-dependent gene expression in human endothelium, increasing cardiovascular risk [74]. Furthermore, the ursodeoxycholic-acid-induced suppression of IFN-γ and CX3CL1 production attenuates the chemotactic and adhesive abilities of liver-infiltrating T cells in primary biliary cholangitis [75]. In short, viral infection is related to the expression of CX3CL1. In the following sections, we reviewed the literature on the changes in CX3CL1 during viral infection and its role in the disease.

## 3. CX3CL1 and Its Receptor in Viral Infection and Associated Diseases

### 3.1. CX3CL1 and Its Receptor in HIV Infection

Human immunodeficiency virus type 1 (HIV-1), which is the causative agent of acquired immunodeficiency syndrome (AIDS), has been known for more than four decades [76]. AIDS remains a major infectious disease threat to global public health. In 1998, CX3CR1 was identified as a fusion co-receptor of CX3CL1 and HIV-1 [77]. In patients with the homozygous CX3CR1-I249M280, a variant haplotype of isoleucine-249 and methionine-280, CX3CL1 binding is reduced, and the progression to AIDS is accelerated [18]. Thus, CX3CR1 is a key recessive genetic risk factor for HIV/AIDS [78]. Table 1 lists the changes in CX3CL1/CX3CR1 in various viral infections.

Mariangela Cavarelli et al. disclosed a novel function of CX3CR1^+^ DCs in the early stages of HIV/simian immunodeficiency virus (SIV) transmission. It seemed that CX3CR1^+^ DCs accumulated in the drainage lymph nodes, while Mφs remained in place during the transition from the CX3CR1^high^ phenotype of tissue resident to the pro-inflammatory CX3CR1^low^ phenotype [79]. It is suggested that SIV infection can cause a rapid shift from CX3CR1^high^ to CX3CR1^low^ in Mφs in the colonic mucosa of macaques, possibly to identify recently recruited cells in the intestine. During HIV-1/Treponema pallidum co-infection, compared with the healthy control group, the density of CX3CR1 was increased in all three monocyte subsets; the increase in CX3CR1 expression on monocytes indicates the presence of systemic inflammation during HIV-1/Treponema pallidum co-infection [80]. 

Ongoing inflammation and the associated complications cause an increase in HIV-1-associated neurological diseases (HAND), including HIV-dementia [81,82]. In the CNS, sCX3CL1 dysregulation in the brain was observed during HIV infection [83]; CX3CL1 was up-regulated in the brain tissue and cerebrospinal fluid of HAND patients and released in response to proinflammatory stimuli, mainly in the neurons, and in co-cultures of astrocytes and HIV-infected Mφs [82,84]. Based on these findings, the mechanism by which HIV-1 mediates the disruption of the CX3CL1/CX3CR1 axis was investigated. It was found that the expression of CX3CR1 in microglia was inhibited by the HIV-1 Tat protein via the NF-κB-yy1 pathway in microglia, attenuating the functional response of microglia induced by CX3CL1 [85]. In addition, CX3CL1/CX3CR1 may mediate HIV-1 envelope protein gp120 neurotoxicity and suppress gp120-induced apoptosis in hippocampal neurons [86]. Table 1 lists the changes in CX3CL1/CX3CR1 in various viral infections.

**Table 1:** Expression of CX3CL1/CX3CR1 in viral infection and associated diseases.

Virus	Cell/Tissue/ Organization	CX3CL1 Expression Status	CX3CR1 Expression Status	Refs.
HIV	Neurons, monocyte, DCs, Mφs	up	up	[82,83,84,87,88,89]
SARS-CoV-2	serum sample	up		[90,91]
Influenza strain (H1N1)	hippocampus	down		[92]
Influenza strain H5N1 and H9N2	DF-1 cell line of chicken embryo fibroblasts lung tissues of chicken	up		[93,94]
RSV	human airway epithelial cells and airway ciliated cells	up	up	[95,96]
CMV	CMV-specific CD8 or effector CD8 T cells		up	[97]
HTNV	nonclassical and intermediate monocyte subsets		up	[98]
CVB3	left ventricle		up	[99]

DCs, dendritic cells; Mφs, macrophages; RSV, respiratory syncytial virus; CMV, cytomegalovirus; HTNV, hantaan virus; CVB3, Coxsackievirus B3.

The expression and function of CX3CR1 on T lymphocytes in HIV-infected patients have also been investigated. Compared with normal individuals, the frequency of CD8 cells expressing CX3CR1 was increased, and was correlated with disease progression in HIV-infected patients [100]. CX3CR1 was expressed on activated and differentiated CCR7- CD45RA^-^ memory lymphocytes and served as the main homing receptor. After binding to its ligand CX3CL1, it participated in the specific migratory pattern of late-stage differentiated CD8 cells and regulated the effector function of CD8 lymphocytes during HIV infection [100,101]. Additionally, platelet interactions can modulate the inflammatory function of CX3CR1^+^CD8^+^ T cells in HIV infection [101]. The role of CX3CL1 in viral infections and related diseases is summarized in Table 2.

Based on these studies and the hypothesis that the transmission of HIV-1 infection in humans is caused by the CX3CL1 trafficking of infected lymphocytes, the following possible immunological methods for preventing and treating HIV-1/AIDS patients are proposed: developing a canarypox-protein HIV vaccine regimen (ALVAC-HIV plus AIDSVAX B/E), designing and testing CX3CL1 antagonists, HIV-specific neutralizing monoclonal antibodies, and other new immunotherapeutic strategies for HIV-1 infection [101,109,110,111].

### 3.2. CX3CL1 and Its Receptor in COVID-19

SARS-CoV-2 is the causative agent of coronavirus disease-2019 (COVID-19), which causes severe symptoms of pneumonia [112]. During the stage of COVID-19-associated hyperinflammation, cells are highly activated and produce large amounts of cytokines, chemokines, and other soluble mediators of immune inflammatory responses, commonly referred to as cytokine storms [113,114]. Recently, Selma Rivas-Fuentes et al. have proposed that, during SARS-CoV-2 infection, CX3CL1 could be positively regulated in the endothelium and contribute to the perpetuation of a pro-thrombotic loop [115]. Previous studies have demonstrated that CX3CL1 is cleaved in an inflammatory environment [116,117]. During COVID-19 infection, the levels of CX3CL1 in the serum inflammatory mediators were higher in critically ill patients than those in severe COVID-19 patients. Moreover, the levels of CX3CL1 were associated with the duration of illness in severe COVID-19 [118]. Patients with chronic obstructive pulmonary disease have systemic inflammatory dysregulation driven by several cytokines, including CX3CL1, which are involved in chemokine signaling pathways associated with the response to severe COVID-19 virus infection [91,119,120]. 

The pathogenesis of immune inflammatory reaction is related to the migration of leukocytes to target tissues, which is driven by chemokines such as CX3CL1 [2]. The initial over-expression of CX3CL1 is conducive to the recruitment of CX3CR1^+^ immune cells to the lung, including monocytes and Mφs [40,121], which could create an inflammatory environment and even lead to organ dysfunction [122]. Moreover, this has been demonstrated in other coronaviruses, where the transmission and homing of leukocytes with different patterns of circulating chemokine levels, with lower increases in CX3CL1 and other chemokines, show good prognostic value [123]. Increased levels of cerebrospinal fluid chemokines, including CX3CL1, might facilitate the trafficking of monocytes to the cerebrospinal fluid, and potentially contribute to the development of neurological symptoms in patients with COVID-19 [90]. As reported by Zhu et al., the migration of DCs and monocytes/Mφs may be mediated by CX3CR1 in COVID-19 patients treated with stem cells [124]. 

In addition, a CX3CR1 inhibitor has been developed and is expected to be applied in human clinical trials in the future [125]. Experimental data analysis supports the protective effect of AZD8797 (an allosteric antagonist of CX3CR1) on SARS-CoV-2-induced injury. The CX3CL1/CX3CR1 signaling pathway may provide a promising target for reducing the neural impact of SARS-CoV-2 [126]. CX3CR1 is one of the potential genes associated with COVID-19 and comorbidity, which provides a basis for further guiding drug and vaccine development to improve treatment efficacy and the development of personalized treatments [127]. 

### 3.3. CX3CL1 and Its Receptor in Influenza

Influenza is a common disease that has been reported in the human population many times over past centuries, sometimes with devastating consequences [128]. The recently emerging and re-emerging strains are the culprits of seasonal and occasional epidemics and pose a serious threat to global public health systems [129]. Influenza virus infections are characterized by the infiltration of leukocytes into infected tissues, especially monocytes. Since pro-inflammatory cytokines lack chemotaxis activity, researchers have focused their interest on members of the chemokine superfamily [130]. The chemokines (CCL4, CCL19, CCL10, and CX3CL1) were upregulated in highly pathogenic avian influenza H5N1 (A/duck/India/02CA10/ 2011)-infected lung tissues of chickens, which may be the key factors determining the severity and outcome of influenza infection in chickens [94,131]. In the convalescent phase, cytokines including CX3CL1 and CD200 are still highly expressed in the brain [15,93]. At this stage, the weight and mobility of the infected mice were completely restored, while the emotional disorders, spatial learning, and memory abilities did not return to normal. This effect may be due to the delayed damage caused by non-neurotic influenza infection involving the aforementioned cytokines [93]. 

Nevertheless, some research results indicate that influenza A/PR/8/34 (H1N1) virus infection can reduce the expression of CX3CL1 in mice hippocampus [92]. When the mutants such as H1N1, H3N2, and H5N1 infected human tracheobronchial epithelial cells, the expression of CX3CL1 was respectively high, showing a certain degree of increase, and undetectable [131]. The reduction in or the loss of CX3CL1 during influenza infection may lead to the impairment of both glial regulation and cognitive function. The previous environmental enrichment-induced increase in CX3CL1 may lay the foundation for limiting the induction of neuroinflammation and better maintaining neuronal structure and synaptic plasticity during influenza virus infection [92]. The different changes in CX3CL1 expression in influenza infection might be associated with the diverse responses of different types of cells to the infection of different subtypes of influenza A virus; the mechanism of the downregulation of CX3CL1 expression caused by H1N1 infecting the hippocampus still needs to be further explored. Siran Lin et al. were the first research group to use a statistical model trained with high-throughput expression data in influenza [132]. After comprehensively analyzing 180 samples from the GEO dataset, a risk score model involving six genes (*CX3CR1*, *KLRD1*, *MMP8*, *PRTN3*, *RETN*, and *SCD*) was established. They found that the expression of CX3CR1 was inversely related to H1N1 disease severity [132,133]. Virus-specific memory CX3CR1^+^CD8^+^T cells are increased during infection, but only a small number are present in the chronic infected state [134,135]. Pulmonary CX3CR1^high^ T cells produce interferon gamma to limit early viral infection in an antigen-independent manner, enhancing the long-term antibacterial activity of alveolar Mφs [136]. Moreover, glucocorticoid-induced TNFR-related protein (GITR) contributes to the accumulation of differentiated effector cells, including CD8^+^ T cell subsets defined by CX3CR1 and Ly6C expression, as well as memory precursors, but there are some differences between subsets [137]. 

Influenza-A-virus-induced mouse pneumonia is a common model for studying the effects of aging on pneumonia-induced muscle function [138]. In young mice, after influenza A infection, the population of tissue-resident Mφs expressing CX3CR1 in skeletal muscle expands without the recruitment of monocytes from the bone marrow. This was followed by the proliferation of muscle satellite cells. Further experiments showed that the phagocytic function of tissue-resident Mφs in the skeletal muscle of older mice was lost. These findings suggest that the signaling induced by phagocytosis in CX3CR1^+^ tissue-resident skeletal muscle Mφs is necessary for the proliferation of satellite cells during muscle recovery after influenza-A-virus-induced pneumonia [130,139]. Vaccination and antiviral therapy are the foundational approaches to limiting the public health impact of influenza [140]. Based on the role of CX3CL1/CX3CR1 in influenza, rational immunotherapy is becoming a promising strategy for improving the outcomes of influenza virus infection.

### 3.4. CX3CL1 and Its Receptor in Respiratory Syncytial Virus Infection

Respiratory syncytial virus (RSV) is a top cause of severe pneumonia in infants and the most common cause of acute lower respiratory infection in young children [141,142]. Among adults, RSV infection produces a wide range of clinical symptoms similar to those of influenza virus infection [143,144]. The two RSV surface proteins, fusion glycoprotein (F protein) and glycoprotein (G protein), are key factors in RSV attachment and entry into cells. They bind to cell-surface heparin sulfate proteoglycans via their heparin-binding domains, thereby inducing protective host immune responses [145]. Previous work found that the G protein also has a CX3C chemokine motif (amino acids 182–186) that facilitates RSV attachment to susceptible cells expressing CX3CR1, to infect primary airway cultures [146,147,148]. CX3CL1 mimicry has been shown to promote RSV infection and alter CX3CL1-mediated chemotaxis of human, cotton rat, and mouse leukocytes [146,149,150,151].

Tatiana Chirkova et al. studied the role of CX3CR1 through mutation in the RSV CX3C motif during RSV infection [95]. Imaging flow cytometry and RSV attachment assay showed that CX3CR1, expressed on airway ciliated cells, interacts with RSV G protein, facilitating virus attachment and the infection of human airway epithelial cells, and modulates cell responses to infection [95,96]. In addition, studies suggest that the interaction of CX3CR1 engagement with the RSV G protein CX3C motif results in intercellular signaling and nucleolin expression, although its role in virus attachment and fusion in RSV infection is still being determined [152]. Dania Zhivaki et al. found that after the binding of surface Ig on neonatal Breg (nBreg) cells, RSV induces the upregulation of CX3CR1 and activates nBreg cells, which results in IL-10 production through the binding of G protein and CX3CR1. In the presence of the CX3CL1, RSV infection was strongly decreased, concomitant with the inhibition of IL-10 secretion in nBreg cells [149] and the decrease in pulmonary inflammation in RSV-infected mice [153]. 

However, compared to wild-type (WT) mice, RSV infection in CX3CR1-deficient (*CX3CR1*^−/−^) neonatal mice resulted in significantly greater neutrophil inflammation in the lungs, accompanied by increased mucus production [154]. A similar study showed that infants carrying a specific I249 M280 CX3CR1 mutation experience more severe bronchiolitis after RSV infection than those without this mutation [95]. These diverse observations highlight the need for further study of host–viral interactions that cause severe disease in infants infected with RSV.

### 3.5. CX3CL1 and Its Receptor in Cytomegalovirus Infection

It was reported that “human cytomegalovirus (HCMV) encodes G-protein-coupled receptors (GPCRs) US28 and US27, which facilitate viral pathogenesis through the engagement of host G proteins”, destroying the host’s immunity [155]. CX3CR1 promotes efficient cell capture when bound to mCX3CL1, while CMV US28 increases cell migration when bound to the same ligand [156]. In experimental animal models, the researchers investigated whether CMV-specific cells in lymph nodes were as abundant as they are in peripheral blood. An interesting phenomenon was observed: CX3CR1 transcripts were highly present at the peak response and remained detectable in the latency stage, while the expression of CX3CR1 was not induced on EBV-specific CD8^+^ T cells or influenza-virus-reactive T cells obtained from a healthy donor [157]. Therefore, in both acute and latent infection, CX3CR1 appears to be a discriminative marker for CMV-specific effector cells. Upon activation of these effector CD8 T cells, they migrate from the lymphatic compartment to the site of inflammation, where they adhere to endothelial cells and extravasate into inflamed tissues [158,159]. Nicole E. Winchester et al. found that CMV infection facilitates the costimulation of CX3CR1^+^CD57^+^CD28^−^CD8 T cells in HIV infection and atherosclerosis via the CD2–LFA-3 axis [160].

In response to murine CMV infection, circulating NK cells were found to be recruited to the salivary glands in a CX3CR1-dependent manner, and then they formed a long-lived, memory-like, natural killer cell, tissue-resident population that suppresses autoimmunity through the TRAIL-dependent elimination of CD4^+^ T cells [161]. Among individuals with HIV, CX3CR1^+^, GPR56^+^, CD57^+^T, and CD4^+^ T cells are often CMV-specific and are associated with diabetes, coronary arterial calcium, and non-alcoholic fatty liver disease [162]. There is evidence that CMV-specific CD4^+^ T cells have been shown to cause endothelial damage in the presence of viral antigens, and the higher the frequency of CMV-specific CD4^+^ T cells, the greater the injury that occurred in the donors [163,164,165]. The reason for the injury is the production of CX3CL1 induced by endothelial cells with the release of IFN-γ and TNF-α from T cells [166]. Moreover, CX3CL1–CX3CR1 interactions play an important role in recruiting NK cells and Mφs and mediate endothelial injury. The specific antibodies against CX3CR1 significantly reduce the chemoattraction of CX3CR1^+^ cells and prevent endothelial damage in CMV infection [165]. Accordingly, they hypothesized that CMV-specific CD8 T cells expressing CX3CR1 have the ability to migrate to the inflamed vascular endothelium. The endothelial-expressed lymph node homing receptor CX3CR1 is an important cell population in individuals with HIV/CMV co-infection, which could promote tumor and viral clearance and may provide a source of cells that respond to immunotherapies in the future [97,134,167,168,169]. 

### 3.6. CX3CL1 and Its Receptor in Other Viral Diseases

DENV-specific CD4^+^ T cells significantly up-regulate CX3CR1, elicit highly polarized states, and mediate direct cytotoxic activity [170,171]. The expression of CX3CR1 on CD4 and CD8 T cells is similar after induction by DENV, ZIKV, and HBV infections, as well as DENV/ZIKV co-infections, which facilitates the regulation of viral processes by precisely controlling inflammatory cells that target the affected tissue [172,173,174]. During DENV and Japanese encephalitis virus infections, large numbers of CD11b^+^ Ly6C^hi^ CCR2^hi^ CX3CR1^low^ inflammatory monocytes infiltrate the liver [175]. It has been demonstrated that CX3CR1 knockout exacerbates Coxsackievirus B3-induced myocarditis [99]. Moreover, the CX3CR1–CX3CL1 axis plays a key role in mediating the transmission of infectious genomic RNA in the pathogenesis of Japanese encephalitis virus [176], as well as in those with hemorrhagic fever with renal syndrome (HFRS), and the expression of CX3CR1 on non-classical and intermediate monocyte subsets may offer new insights into the role of CX3CL1/CX3CR1 in the pathogenesis of HFRS [98]. 

Additionally, the concentration of CX3CL1 in the serum of HBV patients is significantly correlated with disease prognosis [177]. Following DENV infection, the activation of mast cells causes the production of CX3CL1, which facilitates the recruitment of natural killer (NK) and NKT cells and viral clearance [178]. Elevated plasma CX3CL1 levels are associated with the severity of liver disease in HIV/hepatitis C virus (HCV)-co-infected patients with the HCV genotype-1 [179]. Two forms of CX3CL1 display differential activity in adeno-associated virus-treated CX3CL1 knockout mice; specifically, knocking out CX3CL1 leads to severe cognitive deficits, which can be mitigated by sCX3CL1 treatment, while mCX3CL1 can only partially alleviate them [23]. Under physiological conditions, mCX3CL1 has been shown to play a major role in the recruitment and adhesion of infiltrating leukocytes [23]. sCX3CL1 not only acts as a chemotactic agent involved in cell migration, but also serves as a neuroprotective signaling molecule, mediating the anti-inflammatory activity of CX3CL1 in the brain [23]. Proinflammatory mediators, such as sCX3CL1, which are maintained at or below baseline throughout SEOV infection, may mediate SEOV persistence in the lungs [180]. A novel mechanism of CX3CL1 production has been discovered: rhinovirus 16 infection enhances the cleavage of the allergen protease from the apical epithelial surface to produce active CX3CL1, which may contribute to the synergistic effect of allergen exposure and rhinovirus infection in triggering asthma exacerbation and airway remodeling [181]. The role of the CX3CL1/CX3CR1 signaling pathway in the immune pathogenesis of various diseases will guide the future development of therapeutic agents, particularly viral CX3CR1 antagonists, aimed at preventing or slowing the progression of related diseases [180,182,183]. 

## 4. Conclusions

CX3CL1 is a distinctive chemotactic factor produced and secreted by various cells, including immune cells, endothelial cells, and epithelial cells, with the dual functions of adhesion molecules and chemotactic agents. sCX3CL1 induces the migration of CX3CR1-expressing NK cells, cytotoxic T lymphocytes, and Mφs, while mCX3CL1 captures and enhances the subsequent migration of these cells upon stimulation by other chemokines. The expression level of CX3CL1 is associated with the state of the disease, and its improper expression affects various processes, such as leukocyte recruitment, angiogenesis, cell survival, and cell adhesion. Based on the role of the CX3CL1/CX3CR1 system in various clinical diseases, the CX3CL1/CX3CR1 axis has emerged as a promising potential therapeutic target at the appropriate stage due to its ability to drive inflammation.

## Figures and Tables

**Table 2:** The role of CX3CL1 in viral infection and associated diseases.

Virus	Roles
HIV	A. sCX3CL1 inhibits the apoptosis of hippocampal neurons induced by neurotoxic viral proteins [102].
	B. CX3CL1 is involved in neuronal damage through its interaction with microglia, which secrete proinflammatory cytokines [102].
	C. CX3CL1 promotes the accumulation of DCs in the lymph nodes [103].
SARS-CoV-2	A. CX3CL1 facilitates the recruitment and adhesion of CX3CR1^+^ immune cells to target tissues [90].
	B. levels of CX3CL1 is associated with the duration of illness in severe COVID-19 [91].
Influenza strain H1N1	A. *Cx3cr1*^−/−^ mice showed cell-autonomous microglial neurotoxicity [104].
	B. loss of CX3CL1 may lead to changes in both glial regulation and cognitive function [105].
Influenza strain H5N1 and H9N2	A. CX3CL1 impedes neuron–microglia interactions, increased inflammation, and microglial activation [92].
	B. CX3CL1 is a chemotactic factor that occurs in response to H5N1 infection in chickens [106].
RSV	A. CX3CR1 leads to NF-κB activation and CX3CL1 production, and affects the cellular inflammatory response to RSV infection [107].
CMV	A. CX3CL1 promotes the migration of CX3CR1^+^ CMV-specific CD8 T cells to inflamed vascular endothelium [97].
HTNV	A. CX3CL1 level is associated with the severity of hemorrhagic fever with renal syndrome in humans [98].
CVB3	A. CX3CR1 plays a cardio-protective role in CVB3-infected mice [108].

## Data Availability

No new data were created or analyzed in this study. Data sharing is not applicable to this article.
